# A collagen-binding EGFR antibody fragment targeting tumors with a collagen-rich extracellular matrix

**DOI:** 10.1038/srep18205

**Published:** 2016-02-17

**Authors:** Hui Liang, Xiaoran Li, Bin Wang, Bing Chen, Yannan Zhao, Jie Sun, Yan Zhuang, Jiajia Shi, He Shen, Zhijun Zhang, Jianwu Dai

**Affiliations:** 1 Key Laboratory for Nano-Bio Interface Research, Division of Nanobiomedicine, Suzhou Institute of Nano-Tech and Nano-Bionics, Chinese Academy of Sciences, Suzhou 215123, China; 2 State Key Laboratory of Molecular Developmental Biology, Institute of Genetics and Developmental Biology, Chinese Academy of Sciences, Beijing 100080, China; 3 University of Chinese Academy of Sciences, Beijing 100049, China

## Abstract

Many tumors over-express collagen, which constitutes the physical scaffold of tumor microenvironment. Collagen has been considered to be a target for cancer therapy. The collagen-binding domain (CBD) is a short peptide, which could bind to collagen and achieve the sustained release of CBD-fused proteins in collagen scaffold. Here, a collagen-binding EGFR antibody fragment was designed and expressed for targeting the collagen-rich extracellular matrix in tumors. The antibody fragment (Fab) of cetuximab was fused with CBD (CBD-Fab) and expressed in *Pichia pastoris*. CBD-Fab maintained antigen binding and anti-tumor activity of cetuximab and obtained a collagen-binding ability *in vitro*. The results also showed CBD-Fab was mainly enriched in tumors and had longer retention time in tumors in A431 s.c. xenografts. Furthermore, CBD-Fab showed a similar therapeutic efficacy as cetuximab in A431 xenografts. Although CBD-Fab hasn’t showed better therapeutic effects than cetuximab, its smaller molecular and special target may be applicable as antibody–drug conjugates (ADC) or immunotoxins.

Tumor microenvironment includes stromal cells, non-stromal cells, various soluble factors, and an evolving extracellular matrix (ECM)^1^,^2^. It has been recognized that tumorigenesis is not only involved in tumor cells but also a disease of imbalance, in which tumor microenvironment play crucial roles in tumor progression^3^. Collagen is abundant in many types of tumors, which is the main component of tumor ECM and constructs a microenvironment in the development of tumors. Thus, collagen is a new target for cancer therapy^4^,^5^,^6^.

The dense collagen network in the tumor microenvironment is a barrier to tumor therapy because it reduces drug penetration and efficacy^7^,^8^. Reducing collagen content in tumors was a strategy to improve the penetration and therapeutic efficacy of drugs in tumors^7^. Meanwhile, targeting the tumor environment using anti-collagen antibody-drug conjugates (ADCs) is another strategy to enhance the concentration of drugs in tumors and kill tumor cells^9^,^10^. However, the high molecular weight of ADCs usually limits its penetration into tumor core and has a side effect^9^. Polypeptides which showed potent binding affinity of tumor cells or tumor microenvironment, such as intercellular adhesion molecule-1 (ICAM-1), Arg-Gly-Asp (RGD) and Asn-Gly-Arg (NGR), have already been used as carrier for drug delivery in cancer therapy due to their smaller molecular weight and lower undesirable immunogenicity than ADCs^11^,^12^. Collagen binding domain (CBD) is a short peptide, which showed a specific collagen-binding affinity. In our previous study, CBD fusion proteins also showed a highly collagen-binding affinity and achieved controlled release of fusion proteins in the collagen scaffold^4^,^13^,^14^. Therefore, CBD may be used as carrier to deliver the drugs to tumor microenvironment.

Monoclonal antibody (mAbs) was an effective therapeutic drugs for cancer. Cetuximab (Erbitux/C225), an anti-epidermal growth factor receptor (EGFR) human-mouse chimeric immunoglobulinG_1_ (IgG_1_) antibody has already been approved for the treatment of head and neck cancers and colon cancer^15^. However, the entire antibody has a molecular weight of approximately 150 kDa and thus diffuses poorly from the vascular bed into the solid tumor mass^16^. The antigen-binding fragment (Fab) molecule is a 50 kDa fragment of the 150 kDa IgG_1_ molecule that consists of two polypeptide chains. One of these chains contains the light chain variable and constant domains (VL-CL); the other is a truncated heavy chain containing the variable domain and one constant domain (VH-CH1)^17^. Fab retains the antigen-binding regions and the therapeutic effect of its parent antibody^18^,^19^. Furthermore, this fragment demonstrates faster tissue/tumor penetration than the whole antibody^20^. However, the rapidly cleared from the body of Fab may reduce its therapeutic effect. CBD had enhanced the collagen-binding ability and achieved the sustained release of the fusion protein in collagen scaffold^21^. So we concluded that the recombinant protein of CBD and Fab of cetuximab should show a collagen binding capacity, a more specific target, a faster tumor penetration and demonstrated a similar therapeutic activity as whole antibody cetuximab.

In this study, we designed and constructed a collagen-binding EGFR antibody fragment for targeting collagen in tumors. The Fab of cetuximab was fused to CBD and expressed in the methylotrophic yeast *Pichia pastoris*. CBD-Fab specifically bound to collagen and maintained the antitumor activity of cetuximab both *in vitro* and *in vivo*. Early treatment with CBD-Fab prevented tumor formation and significantly suppressed the growth of tumors in A431 xenografts.

## Results

### CBD-Fab and NAT-Fab antibody fragment expression, production and purification

**Figure 1:**
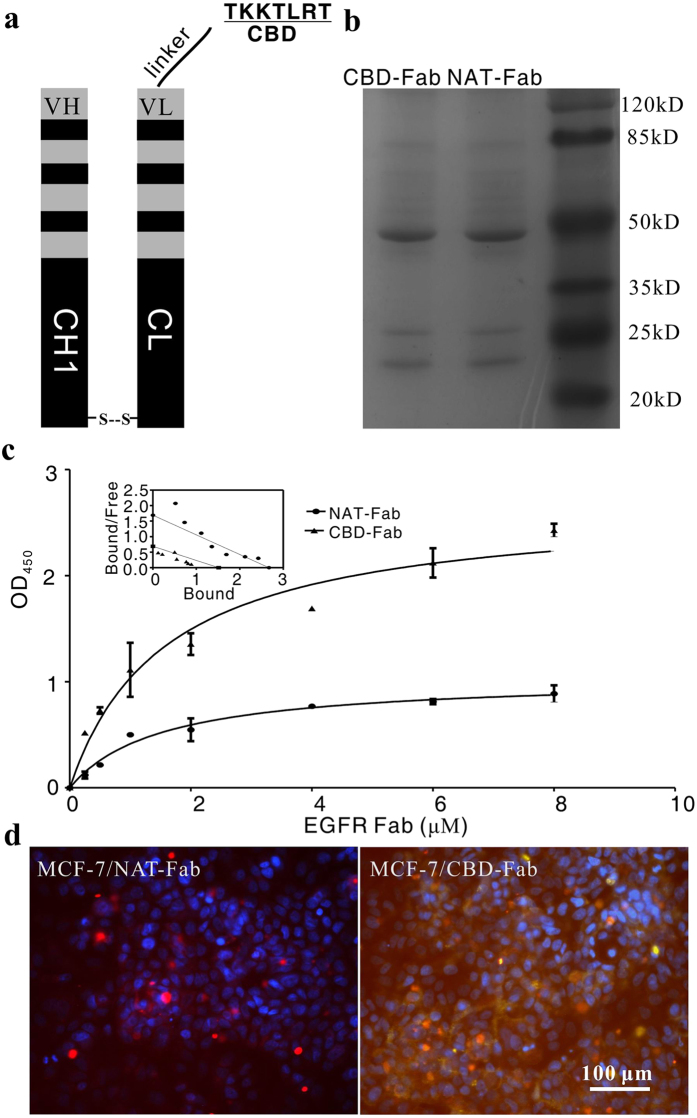
Purification and collagen-binding of CBD-Fab *in vitro*. (**a**) Diagrams of CBD-Fab. (**b**) SDS-PAGE analysis of CBD-Fab and NAT-Fab. (**c**) Collagen-binding assay of CBD-Fab. *K*_d_ values for the collagen-binding of CBD-Fab and NAT-Fab were analyzed by Scatchard analysis. *K*_d_ values of NAT-Fab and CBD-Fab were 1.58 μM and 0.21 μM, respectively. (**d**) The collagen-binding of NAT-Fab and CBD- Fab in the ECM of MCF-7 *in vitro.* MCF-7 cells stained with CBD-Fab or NAT-Fab or anti-type I collagen antibody. Nuclei were stained with DAPI.

The single *P. pastoris* colonies which integrated the linearized pPICZα-B-CBD-Fab-L and pPIC9K-Fab-H were analyzed by colony PCR. Ten positive colonies (Mut+) were confirmed using a small-scale expression trial. The supernatants were collected and analyzed via 15% SDS-PAGE. The recombinant CBD-Fab fragment was purified by Ni-NTA affinity chromatography. Under the optimized conditions, about 2 mg CBD-Fab antibody fragment with 90% purity was obtained from 1 L supernatant by nickel affinity chromatography (Fig. 1a,b). The Fab without CBD (Natural-Fab, NAT-Fab) was prepared following the same method as CBD-Fab.

### Specific collagen-binding of CBD-Fab *in vitro*

The collagen binding of CBD-Fab and NAT-Fab was showed using modified ELISA. As showed in Fig. 1c, the OD_450_ in the CBD-Fab group was higher than that of the NAT-Fab at each concentration. The dissociation constant (*K*_d_) values of NAT-Fab and CBD-Fab for collagen were 1.58 μM and 0.21 μM, respectively (Fig. 1c), as calculated using Scatchard analysis. The lower *K*_d_ value indicated higher affinity of CBD-Fab to collagen than NAT-Fab. MCF-7 (EGFR-) was cultured to further prove the collagen-binding of CBD-Fab *in vitro*. Immunofluorescence showed that CBD-Fab specifically bound to collagen in the ECM of MCF-7 (Fig. 1d). These results clearly demonstrated that CBD-Fab had a specific collagen-binding capacity *in vitro.*

### EGFR-Binding characteristic of CBD-Fab and NAT-Fab antibody fragments

**Figure 2:**
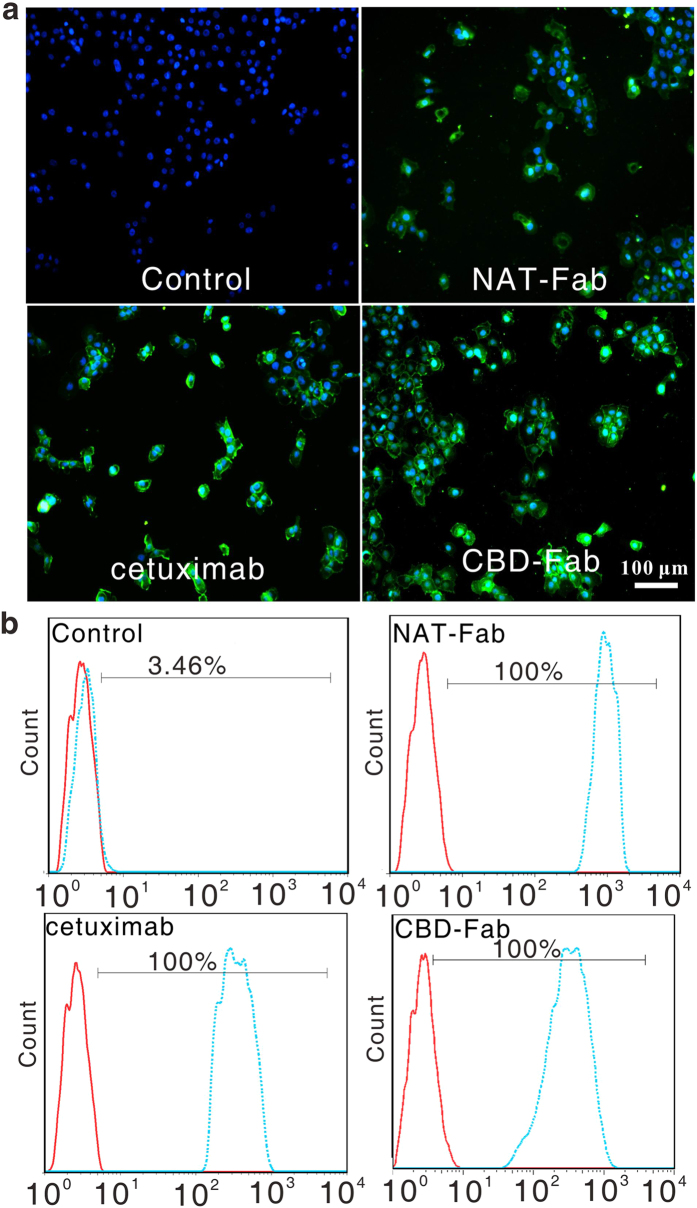
EGFR-Binding of CBD-Fab and NAT-Fab. (**a**) Immunofluorescence of the binding of CBD-Fab and NAT-Fab to EGFR on A431 cells. (**b**) The EGFR-binding of CBD-Fab and NAT-Fab were analyzed via flow cytometry. Histograms were generated using FlowJo software (Tree Star, Inc., San Carlos, CA). The dashed lines represent the cells incubated with antibody, and the solid lines refer to the negative control.

The Fab fragment should retain antigen binding domain of parent of antibody. Firstly, we investigated the EGFR-binding of CBD-Fab and NAT-Fab using immunocytochemistry and flow cytometry. The immunocytochemistry results showed 1 μg/mL CBD-Fab and NAT-Fab displayed specific EGFR-binding activity on the surface of A431 cells (Fig. 2a). The flow cytometry were used to further analyze the EGFR-binding of CBD-Fab and NAT-Fab. The results showed A431 cells displayed 100% FITC fluorescence ratio after incubated with 1 μg/mL CBD-Fab, NAT-Fab, or cetuximab (Fig. 2b). The results showed that CBD-Fab or NAT-Fab and cetuximab had a similar EGFR-binding characteristic *in vitro*. We can demonstrate that the recombinant CBD-Fab and NAT-Fab maintained the EGFR-binding ability of cetuximab.

### Antitumor activity of CBD-Fab and NAT-Fab *in vitro*

**Figure 3:**
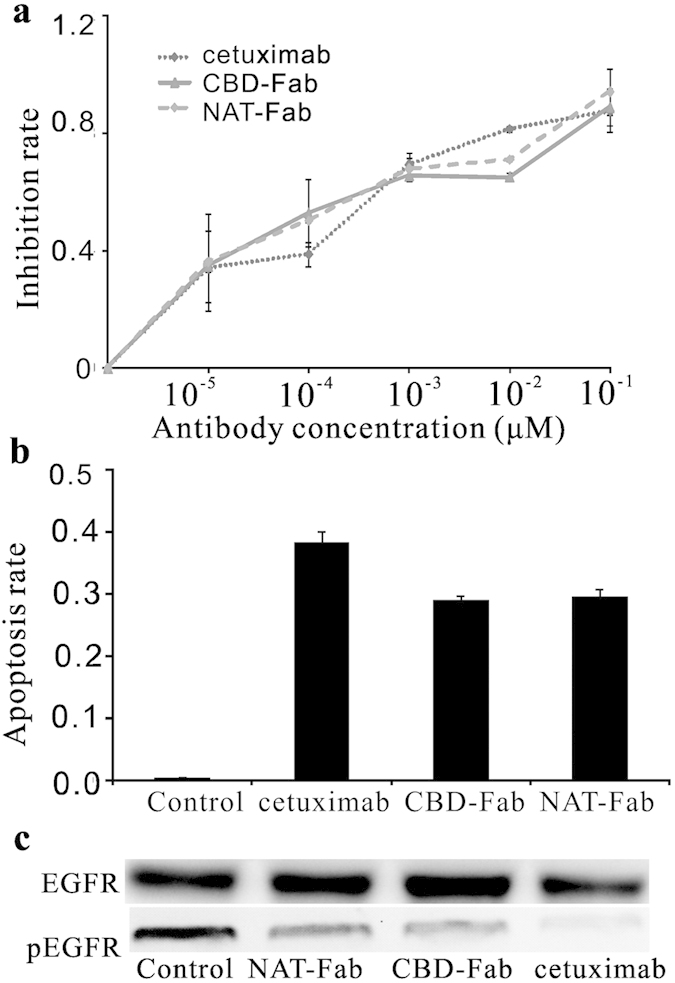
The anti-tumor activity of CBD-Fab and NAT-Fab *in vitro.* (**a**) A431 cells were treated with CBD-Fab, NAT-Fab, or cetuximab at indicated doses for 48 h and then the cell proliferation was checked by MTT assay. Data were from one of the independent experiments performed in triplicate. (**b**) The Annexin V staining was used to check the apoptosis of A431 cells induced by CBD-Fab, NAT-Fab, or cetuximab. The data represent one independent experiment performed in triplicate. Values were presented as the mean ± SD of three independent measurements. (**c**) Western blot analysis for phosphorylated EGFR in A431 cells exposed to different concentrations of antibody. The A431 cells were incubated with 10^−1^ μM EGFR-specific mAbs (cetuximab, CBD-Fab, or NAT-Fab) and stimulated with 20 ng/mL EGF. The phosphorylation of EGFR in A431 was analyzed by immunoblotting. The experiments were repeated at least twice.

We performed MTT assay to determine the inhibiting efficacy of CBD-Fab and NAT-Fab to the growth of A431 cells. The inhibition rate of CBD-Fab and NAT-Fab to A431 cells were showed in Fig. 3a and the IC_50_ of CBD-Fab and NAT-Fab were similar (approximately 1.68 × 10^−4^ μM). Annexin V-FITC/PI assay results showed that CBD-Fab and NAT-Fab primarily induced early apoptosis in A431 cells after treated with 1.68 × 10^−4^ μM CBD-Fab or NAT-Fab for 48 h (Fig. 3b). The apoptosis rate of the A431 cells treated with 1.68 × 10^−4^ μM CBD-Fab, NAT-Fab, and cetuximab for 48 h was 29.6% ± 5.4%, 29.1% ± 13.7% and 38.3% ± 17.1%, respectively (Fig. 3c). The apoptosis rate of A431 cells treated with CBD-Fab and NAT-Fab had no significant difference. These results indicated that CBD-Fab and NAT-Fab had a similar anti-tumor activity as cetuximab.

### The EGFR phosphorylation inhibition of CBD-Fab and NAT-Fab

The proliferation of tumor was associated with the levels of activated EGFR. We used immunoblotting to show the ability of CBD-Fab or NAT-Fab to block ligand-induced receptor phosphorylation in the presence of EGF. As shown in Fig. 3c, incubation of A431 cells with the antibodies resulted in a significant down-regulation on EGFR phosphorylation in all treatments. CBD-Fab or NAT-Fab showed a similar inhibition activity of ligand binding as cetuximab.

### The biodistribution and sustained release of CBD-Fab *in vivo*

**Figure 4:**
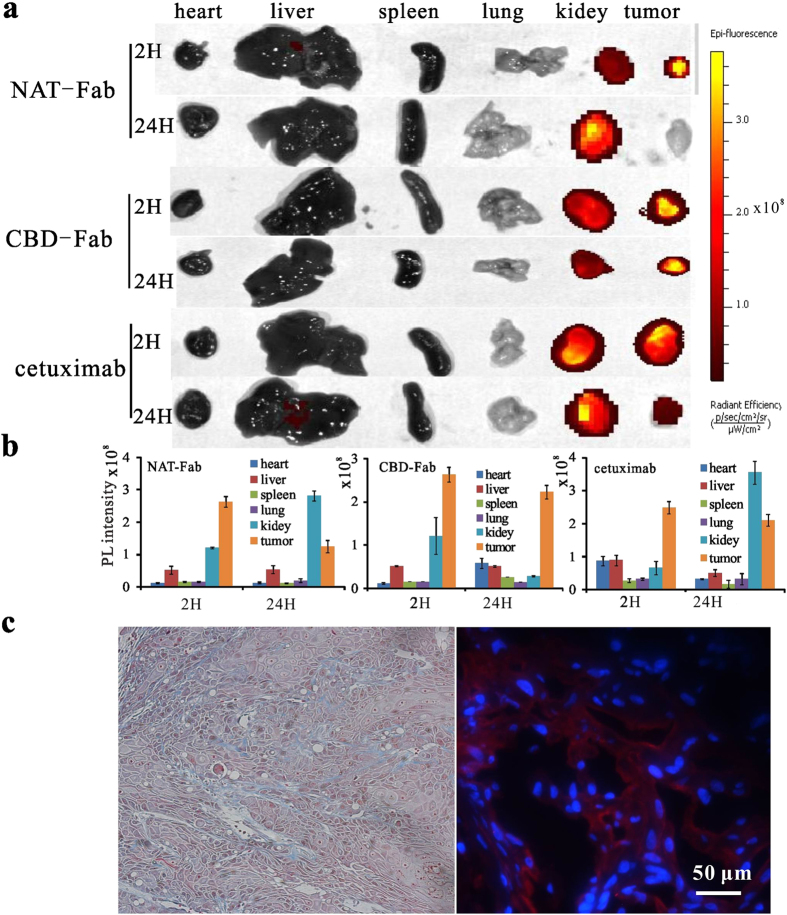
The biodistribution of different antibody *in vivo* and the abundant collagen in tumors. (**a**) The photographs of heart, liver, spleen, lung, kidney and tumor under NIR illumination clearly demonstrated the changes of antibody concentration in these organs. (**b**) PL intensities of liver, spleen, kidney, lung, heart, and tumor indicated the antibody concentrations in these organs collected at different time points. Values represented means ± SD, n = 3. (**c**) Masson’s trichrome staining of A431 tumor sections were performed to show the collagens in the ECM of A431 tumors (Left). The collagen fibers were stained blue, the nuclei were stained black and the muscle or erythrocytes were stained red. Immunofluorescence was further performed to show the collagen in tumor tissue (Right). Anti- type I collagen antibody was used to stain collagen in tumors, the nuclei were stained with DAPI.

We firstly investigated the biodistribution of CBD-Fab, NAT-Fab, or cetuximab in different organs in xenografts. The mice were intraperitoneally (i.p.) injected with Cy7-labeled antibody (15 μM in 450 μL), and then sacrificed at 2, 12 and 24 h, respectively. The organs of mice were harvested and imaged under irradiation at 710 nm. The results showed the fluorescence intensity of the organs for a semi-quantitative biodistribution analysis, suggested that the CBD-Fab mainly enriched in tumors. As showed in Fig. 4a,b, CBD-Fab was quickly transported into the tumors and showed a slowly decrease in tumors at 2 h and 24 h. While NAT-Fab showed a quickly transport to the tumors at 2 h, while mainly accumulated in kidneys after 24 h and leaked from the kidneys very quickly.

Collagen constituted the physical scaffold of tumor microenvironment. Masson’s trichrome staining and immunofluorescence analysis was performed to delineate the collagen network around the tumor tissue. Masson’s trichrome staining showed there were distinct blue collagen fibers in tumor xenograft tissue (Fig. 4c Left). Anti type I collagen antibody was further used to show collagen in tumors. The immunofluorescence analysis also showed the abundant collagen in tumors (Fig. 4c Right). Thus, collagen is a universal part of the ECM in A431 xenografts.

**Figure 5:**
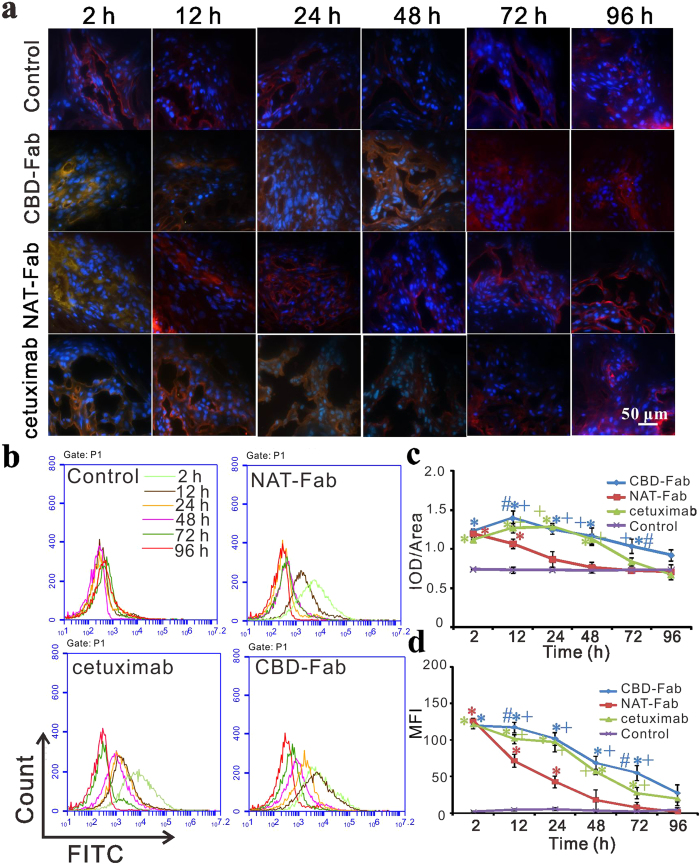
Sustained release of CBD-Fab *in vivo*. (**a,c**) Immunohistochemistry and the integrated optical density (IOD)/Area of FITC positivity in images. Cryosections of tumors from the tumor xenografts at 2, 12, 24, 48, 72, and 96 h after injection of the drugs was performed to detect the remaining of CBD-Fab, NAT-Fab, and cetuximab (green). The collagen in tumors was co-stained with anti-collagen antibody (red). The nuclei were stained with DAPI (blue).The IOD/Area of FITC positivity in images was analyzed with Image-Pro Plus analysis software (Media Cybernetics, Inc., Silver Spring, MD, USA) and calculated in each image. At least 5 images from each analysis were used for quantification and statistical analysis. (**b,d**) Histograms of flow cytometry (**b**) and the mean fluorescent intensity (MFI) of flow cytometry were showed in diagram (**d**).The remain of drugs in different group (n = 5) were tested by flow cytometry C6. Every experiment was repeated three times. Statistical analyses were performed using one-way ANOVA.* indicated *p* < 0.05 for samples compared with control. + indicated *p* < 0.05 for samples compared with NAT-Fab. # indicated *p* < 0.05 for samples compared with cetuximab.

Then flow cytometry and immunohistochemistry were used to detect the remains of CBD-Fab and NAT-Fab in tumors at different time points. Immunohistochemistry was performed to confirm the retention time of CBD-Fab was longer than that of NAT-Fab and cetuximab *in vivo* (Fig. 5a). As showed in Fig. 5c, the IOD/Area value of the CBD-Fab group decreased more slowly than that of the NAT-Fab and cetuximab groups at each time point (Fig. 5c). We can see that CBD-Fab and NAT-Fab targeted faster into tumors than cetuximab at 2 h, and NAT-Fab group had a rapider decrease than CBD-Fab group (Fig. 5c). Flow cytometry analysis also showed CBD-Fab had a longer retention time than NAT-Fab and cetuximab in tumors. The mean fluorescence intensity (MFI) of CBD-Fab was higher than NAT-Fab group and the cetuximab group at each time point. After the injection of each drug for 96 h, the MFI of tumor cells in the CBD-Fab group was 27.3 but only 1.71 and 19.5 in the NAT-Fab and cetuximab groups, respectively (Fig. 5b,d). These results demonstrated that CBD enhanced the binding of Fab to collagen in tumors and had a longer retention time in tumors compared with NAT-Fab and cetuximab.

### Preventive efficacy of CBD-Fab in A431 s.c. xenografts

**Figure 6:**
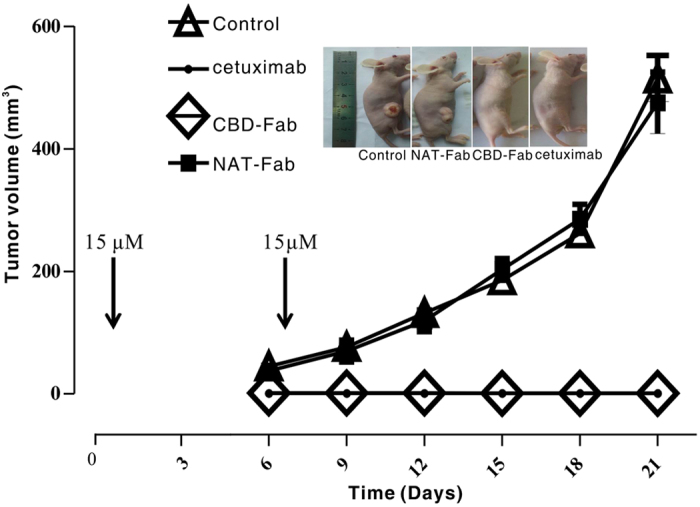
Early treatment with CBD-Fab, NAT-Fab, and cetuximab prevented tumor growth. Five mice per group were injected s. c. with 5 × 10^6^ A431 cells, and 15 μM CBD-Fab, NAT-Fab, and cetuximab in 450 μL PBS were administered on days 1 and 7 after tumor inoculation. The tumor volumes were monitored every two days starting on day 1.

Many malignancies are associated with the accumulation of fibrillar collagen types I and III, as well as the degradation of collagen type IV. Next, we sought to determine whether CBD-Fab was enriched around the collagen scaffold of tumors and inhibited tumor growth. Mice were treated i.p. with 15 μM CBD-Fab, NAT-Fab, and cetuximab in 450 μL on day 1 and day 7 after tumor induction (early treatment). The tumors treated with PBS and NAT-Fab reached a volume of approximately 500 mm^3^ by day 21, whereas the volumes of the CBD-Fab- and cetuximab-treated tumors was less than 30 mm^3^ (Fig. 6). Moreover, the mice treated with CBD-Fab and cetuximab were fully protected against tumor growth.

### Therapeutic efficacy of CBD-Fab in established A431 xenografts

**Figure 7:**
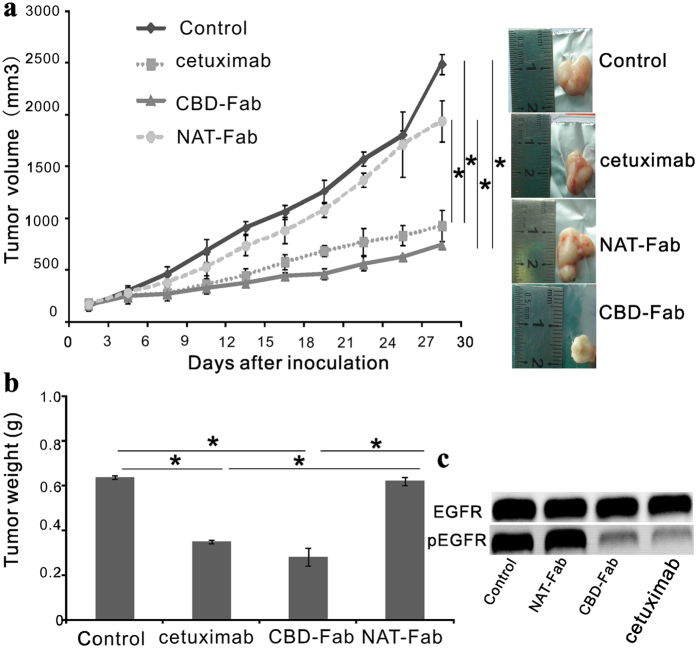
Effect of CBD-Fab, NAT-Fab, and cetuximab on established A431 tumor xenografts. When the tumors reached an average volume of 200 mm^3^, the mice were randomly allocated to four groups (n = 5), and 15 μM CBD-Fab, NAT-Fab, and cetuximab in 450 μL PBS were injected i.p. every two days for 4 weeks. The control mice were treated with the same volume of PBS. (**a**) Tumor volume was measured twice weekly via caliper measurements. Tumor volume was calculated using the following formula: π/6 × larger diameter × (smaller diameter)^2^. (**b**) The last tumor weight was obtained. (mean ± SD, n = 5). *p < 0.05. (**c**) Western blot analysis for phosphorylated EGFR in A431 tumor tissues. Immunoblot assays were performed to show the phosphorylation of EGFR in tumor tissue after treatment.

Early treatment with CBD-Fab effectively prevented A431 xenograft tumor formation *in vivo*. Next, we investigated the inhibitory effect of CBD-Fab and NAT-Fab on established A431 tumor xenografts. When the tumors reached an average volume of 200 mm^3^, 15 μM NAT-Fab, CBD-Fab, or cetuximab in 450 μL PBS were injected i.p. every two days. The tumor volume was significantly reduced in the CBD-Fab-treated mice on day 30; the tumor volumes of the mice treated with PBS and NAT-Fab were estimated to be 2,489.483 ± 40.315 mm^3^ and 1,940.88 ± 38.48 mm^3^, respectively, whereas the volumes of the CBD-Fab and cetuximab groups were 745.41 ± 39.16 mm^3^ and 880.484 ± 120.632 mm^3^, respectively (Fig. 7a). CBD-Fab inhibited tumor growth more effectively than cetuximab or NAT-Fab. The tumor weight in the CBD-Fab group was significantly lower than the NAT-Fab and cetuximab groups (Fig. 7b). There was also a down-regulation of EGFR phosphorylation in CBD-Fab and cetuximab treatments compared with control and NAT-Fab treatment (Fig. 7c). These results clearly demonstrated that CBD-Fab effectively inhibited tumor growth *in vivo*.

### Immunohistochemical analysis

**Figure 8:**
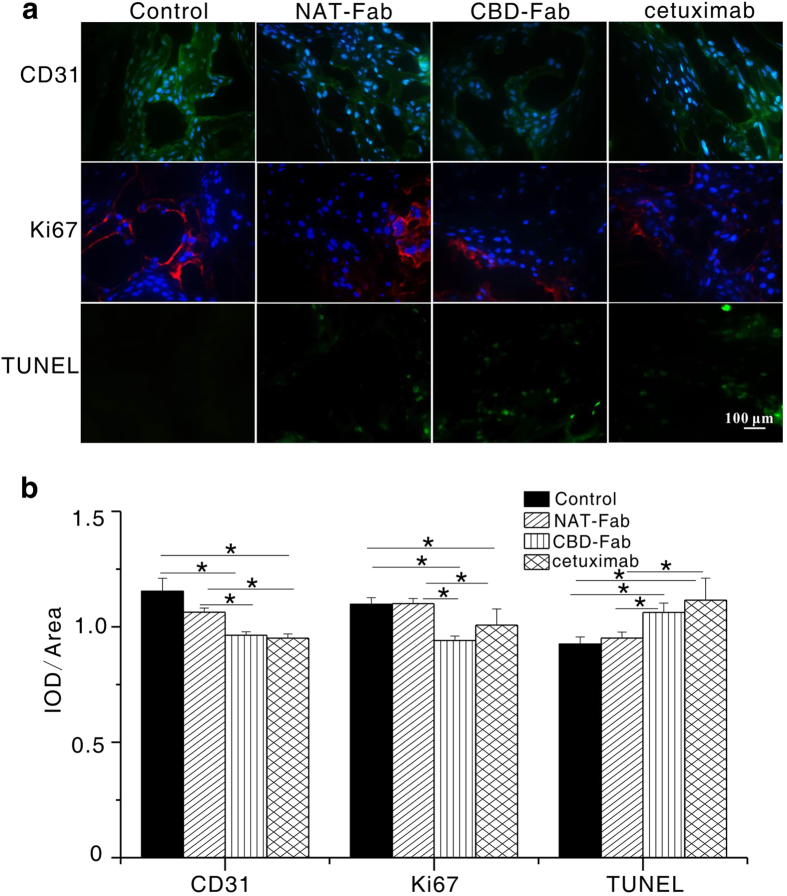
CD31, Ki67, and TUNEL staining of representative tumor sections from different treatment. (**a**) Representative images of tumors treated with 15 μM CBD-Fab, NAT-Fab, and cetuximab in 450 μL PBS for anti-CD31, anti-Ki67, and TUNEL immunostaining. The nuclei of the section stained with CD31 and Ki67 were labeled using DAPI. (**b**) The IOD/Area of anti-CD31, anti-Ki67 and TUNEL positivity were calculated in each image. At least 5 images from each analysis were used for quantification and statistical analysis. (mean ± SD, n = 5). *p < 0.05.

Immunohistochemistry for CD31, Ki-67, and TUNEL were performed using cryosections from tumor xenograft. We investigated the mitotic and apoptosis index of tumors treated CBD-Fab, NAT-Fab or cetuximab by immunostaining tumor sections with anti-Ki67 and TUNEL assays (Fig. 8a). Anti-CD31 immunostaining was also conducted to evaluate microvessel density in different treatment (Fig. 8a). The results showed that the IOD/Area of Ki67 in CBD-Fab group was significantly lower than those in the other groups. It was (0.961 ± 0.028) in the CBD-Fab group, (0.941 ± 0.039) in the cetuximab group, (1.100 ± 0.022) in the NAT-Fab group, and (1.097 ± 0.028) in PBS group (Fig. 8b). The IOD/Area of CD31 was significantly lower in the CBD-Fab group (0.951 ± 0.018) and the cetuximab group (0.956 ± 0.027) compared with the control (1.148 ± 0.088) and NAT-Fab groups (1.063 ± 0.018) (Fig. 8b). TUNEL staining of tumors treated with CBD-Fab showed a significant increase in cell apoptosis than those treated with NAT-Fab or cetuximab. The decrease of tumor growth can be attributed to both decreased cell proliferation and increased cell death. These results indicated that CBD-Fab and cetuximab had a similar therapeutic efficacy and better anti-tumor efficacy than NAT-Fab.

## Discussion

Many previous studies showed that tumor environment is not just involved in transformed cells, but rather, because of the interactions between mesenchymal and epithelial cells that impose reciprocal influences, the microenvironment acts as an active participant throughout cancer initiation, progression, and metastasis^22^,^23^,^24^. Epithelial cell adhesion molecule (EpCAM) was one of component of the tumor microenvironment. Anti EpCAM (scFv)-MAP fusion protein showed a therapeutic potential for the targeted elimination of EpCAM^+^ carcinomas^25^. Collagen, the mainly component of tumor microenvironment, as much as 30% of total mammalian protein mass, which provides a support for tumor cells adhesion and migration^26^,^27^. Anti-collagen antibody had been used as antibody–drug conjugates (ADC) form and achieved sustained release of cytotoxic agent in tumors^9^. Collagen has been recognized as a target for cancer therapy^24^,^28^,^29^. In our study, we used masson’s staining and immunofluorescence analysis to show the abundant collagen in A431 tumors. Furthermore, the biodistribution of CBD-Fab in tumor, the longer retention time, and better therapeutic efficacy of CBD-Fab than cetuximab *in vivo* showed the interaction of CBD and collagen in tumors. These results indicated the potential of collagen as a target for cancer therapy.

Engineered antibodies are widely used for therapeutic applications and account for more than 30% of the biopharmaceuticals in clinical trials^30^,^31^. However, a number of problems associated with diminished antibody efficacy must be addressed. A full-sized antibody slows vascular diffusion and prevents deep penetration into solid tumors^17^. Moreover, radionuclide- or cytotoxin-coupled molecules persist longer in the general circulation, causing toxic side effects. An equally important yet sometimes overlooked issue is the production of sufficient quantities of monoclonal antibodies (mAb). mAb therapy involves high doses (usually more than 1 g per patient per year) and can only be generated in relatively expensive mammalian cells^16^. Fermentor Fab fragments have been expressed due to their small size, fast tissue penetration, ease of genetic manipulation, and low-cost scalable fermentation processes^17^,^18^. We firstly selected *P. pastoris* as a system to express CBD fused to the Fab of cetuximab. Compared with mammalian cell lines, the *P. pastoris* system offered intrinsic advantages, such as ease of genetic manipulation, stable expression, rapid cell growth, and low-cost scalable fermentation processes. As a 7-amino acid peptide, CBD was easily fused to cytotoxic proteins or peptides and expressed in *P. Pastoris*. Moreover, the recombinant proteins were considered inherently safe in contrast with synthetic cytostatic drugs^6^. CBD-Fab displayed similar advantages. The expression level of CBD-Fab in *P. pastoris* reached approximately 2 mg/L in a shake-flask culture. A higher production of the protein may be possible in fed-batch fermentations.

The Fab is a 50 kDa fragment of the 150 kDa IgG_1_ molecule that contains a heavy chain shortened by constant domains CH2 and CH3, and this molecule retains the antigen-binding regions and therapeutic effect of the parent antibody, while which had a faster tumor tissue penetration than parent antibody due to its smaller molecular weight^17^,^18^. The recombinant CBD-Fab retained the EGFR-binding and antitumor activity of cetuximab. Generally, molecules with a molecular weight smaller than 60 kDa *s*howed more rapid biodistribution while clearance too rapidly from the body, resulting in insufficient drug accumulation in tumors^17^,^32^,^33^. In our study, CBD was fused to the N-terminal of Fab-L using a linker, which indeed enhanced the target of CBD-Fab *in vitro* and *in vivo.* CBD-Fab mainly accumulated in tumors and transported into tumor tissues faster than cetuximab due to its smaller molecular weight (Figs 4 and 5). Meanwhile, CBD enhanced the target and retention time of CBD-Fab and achieved its controlled release through dynamically binding to collagen in tumors. The results that CBD-Fab prevented tumor formation as cetuximab and significantly suppressed the growth of A431 xenograft tumors better than cetuximab showed the function of CBD in controlled release of CBD-Fab, although CBD-Fab had a smaller molecular weight than cetuximab and lack of Fc. The dual target of CBD-Fab maintained the function of antibody and got a collagen binding ability.

Few studies have reported the application of collagen-targeting antibodies or proteins in cancer therapy^9^,^34^. Although CBD-Fab did not show better therapeutic effects than cetuximab, its smaller molecular and special target may be applicable as antibody–drug conjugates (ADC) or Immunotoxins. The strategy that CBD-Fab enhanced the target of the drugs and achieved its control release in tissues can be applied to other anticancer agents including molecular targeting agents by minor modification. CBD, as a leader peptide, could achieve the protein or drugs specially target to collagen in tumors or other tissues *in vivo*.

## Methods

### Cell culture

A431 (EGFR positive) and MCF-7 (EGFR negative) cells were purchased from the Cell Bank of the Chinese Academy of Sciences (Shanghai, China). The cell lines were tested and authenticated by short tandem repeat (STR) DNA profiling analysis. The cell lines were immediately expanded and frozen such that they could be restarted every 3 to 4 months from a frozen vial of the same batch of cells. A431 and MCF-7 were maintained at 37 °C in a 5% CO_2_ humidified atmosphere with DMEM medium supplemented with 10% fetal bovine serum, 100 IU/mL penicillin, and 100 μg/mL streptomycin^35^.

### Plasmid construction

The DNA sequences of the entire light chain genes (VL and CL) and heavy chain genes (VH and CH1) of cetuximab were obtained from United States patent US 7, 060, 808^36^ and generated using synthetic oligonucleotides (Genewiz Tech Co., LTD; Suzhou, China). Both the CBD + (G_4_S)_3_ + VL+ CL and VH + CH1 DNA chains of Fab were fused via overlapping PCR (Fig. 1a). Finally, the heavy and light chains of the Fab DNA sequences were inserted into pPICZαB and pPIC9K (Invitrogen, Shanghai, China), respectively. The recombinant plasmids pPICZαB-CBD-Fab-L and pPIC9K-Fab-H were confirmed via restriction analysis and sequencing.

### Transformation of *P. pastoris*

*P. pastoris* wild type strain GS115 was used as the host strain. The recombinant plasmids pPICZαB-CBD-Fab-L and pPIC9K-Fab-H (10 μg) were linearized with *Sal*I and *Bst*XI, respectively, and the linearized plasmids were transformed into *P. pastoris* strain GS115 with an electroporator (Bio-Rad, Shanghai, China) according to the manufacturer’s instructions. pPICZαB-CBD-Fab-L and pPIC9K-Fab-H were integrated into yeast using Zeocin (100 μg/mL) and G418 (1 mg/mL to 4 mg/mL) selections^37^,^38^. The electroporation mixtures were plated on YPDS plates (1% yeast extract, 2% peptone, 2% dextrose, 1 M sorbitol, 2% agar, 100 μg/mL Zeocin, and 1 mg/mL to 4 mg/mL G418) to characterize the methanol-utilizing phenotype. The empty vectors pPICZαB and pPIC9K were similarly linearized and transformed into GS115 as negative controls. The selected strains with the AOX1 promoter were the methanol utilization positive phenotype (Mut+), meaning that they were fully capable of metabolizing methanol as the sole carbon source. The Mut+ strains were obtained from YPDS plates, and the inserts were confirmed via PCR amplification of a yeast genomic DNA template. The specific primers were as follows: Fab-L (F: CTCGAGAAAAGAACCAAGAAGACCTTA, R: GCGGCCGCACACTCTCCCCTGTTGAAGCTC) and Fab-H (F: GAATTCCAGGTGCAGCTGAAACAGAGCGG, R: GCGGCCGCGTGAGTTTTGTCACAAG). Fab- and L- and H-expressing clones were screened via ELISA and SDS-PAGE analyses. These Mut+ strains were then selected for suspension culture. Fab without CBD (NAT-Fab) that was used as a control was prepared using the same method as CBD-Fab.

### Shake flask culture

Single colonies with the Mut+ phenotype were selected and transferred to 50 mL BMGY (1% yeast extract, 2% peptone, 100 mM potassium phosphate (pH 6.0), 1.34% YNB, 4 × 10^−5^% biotin, and 1% glycerol) in 500 mL baffled flasks. Cultures were incubated overnight at 28 °C with vigorous shaking (250 rpm) until OD_600_ = 2. Then, the yeast cells were harvested and resuspended in 200 mL of BMMY induction medium (BMGY with 0.5% methanol instead of glycerol) in 1,000 mL baffled flasks. Maintained induction was achieved by adding 100% methanol pulses every 24 h to a final concentration of 0.5%. After 96 h of culture, the medium was harvested via centrifugation (8,000 × *g*, 15 min), and the supernatant was concentrated 50 times using ultrafiltration and analyzed via 15% SDS-PAGE. Then, the recombinant protein in the concentrated product was purified using affinity chromatography (His-Trap affinity columns, GE).

### Collagen-binding assay of CBD-Fab *in vitro*

The collagen-binding ability of CBD-Fab was tested using a modified ELISA assay as previously described^13^. Briefly, the recombinant proteins CBD-Fab and NAT-Fab at concentrations from 0 μM to 10 μM were added to 96-well plates precoated with collagen and incubated at 4 °C overnight, and then the plates were washed five times to remove the unbound proteins. The remaining proteins were detected by ELISA assay. Anti-histidine polyclonal antibody (1:1000, Sigma Aldrich, Shanghai, China) was used to detect remaining NAT-Fab and CBD- Fab. After three washes as above, 100 μL aliquots of sheep anti-mouse-HRP antibody (1:10, 000, Sigma Aldrich, Shanghai, China) were added and incubated for 1 h at RT, followed by three washes as above. The HRP reaction product was developed by incubation with TMB (Beyotime Institute of Biotechnology, Haimen, China) for 10 min at room temperature. The results were quantified at 450 nm using an ELISA reader (Perkin-Elmer Victor X4, Shanghai, China). The dissociation constant *K*_d_ values of the two proteins to collagen were calculated by GraphPad Prism 5.0. In order to further detect the binding of CBD-Fab to collagen in the ECM of tumor cells, MCF-7 cells (EGFR negative) were seeded in 48-well plates at 20, 000 cells per well and inoculated with NAT-Fab and CBD-Fab respectively. Then the cells were washed five times with PBS and incubated with anti-His mAb (1:2,000, Sigma Aldrich, Shanghai, China) and anti-type I collagen antibody(1:500, BIOSS Inc, Beijing, China) for 2 h. Subsequently, secondary antibody anti-mouse IgG_1_-FITC (1:2,000, Sigma Aldrich, Shanghai, China) and anti-rabbit IgG Fc (DyLight® 594) (1:500, Abcam, Shanghai, China) were incubated with the cells for 1 h. The nuclei were labeled with DAPI (1:2,000, Sigma Aldrich, Shanghai, China). After final washing five times with PBS, the cells were visualized under the confocal microscope (Nikon A1, Tokyo, Japan).

### Binding characterization of EGFR-specific Fab *in vitro*

First, we investigated the binding of CBD-Fab and NAT-Fab to EGFR using immunofluorescence analysis in EGFR-overexpressing A431 cells. A431 cells were seeded in 48-well plates at 10,000 cells per well and treated with CBD-Fab and NAT-Fab^37^. Briefly, A431 cells were washed three times with PBS and incubated with the CBD-Fab and NAT-Fab in 100 μL (1 μg/mL) at 4 °C for 1 h. The cells were washed five times and incubated with anti-His antibody (1:2,000 dilution, Sigma-Aldrich, Shanghai, China) at 37 °C for 2 h. Immunostaining was carried out in the dark with a secondary anti-mouse IgG_1_-FITC for 1 h. The nucleus was labeled with DAPI. After washing five times, the cells were visualized using a confocal microscope (Nikon A1, Tokyo, Japan).

Flow cytometry analysis was used to assess the EGFR-binding ability of CBD-Fab and NAT-Fab. A431 cells (1 × 10^6^ cells) were harvested, washed five times with PBS, and incubated with the CBD-Fab and NAT-Fab in 100 μL PBS (1 μg/mL) at 4 °C for 1 h. Then, the cells were incubated with anti-His antibody at 37 °C for 2 h, followed by anti-mouse IgG_1_-FITC at 37 °C for 30 min. Then, the cells were analyzed using flow cytometry C6 (BD Biosciences, San Jose, CA, USA). The cells treated with cetuximab served as a positive control.

### Cell proliferation assay

Tumor cell growth *in vitro* was evaluated using MTT assays^32^. A431 cells were seeded in 96-well plates at 10,000 cells per well. Then, the cells were incubated with serial dilutions (0 μM, 10^−5^ μM, 10^−4^ μM, 10^−3^ μM, 10^−2^ μM, and 10^−1^ μM) of CBD-Fab, NAT-Fab, cetuximab or control culture medium for 48 h. For the MTT assays, the cells were incubated in medium containing 100 μL 3-(4, 5-dimethylthiazol-2-yl)-2, 5-diphenyltetrazolium bromide (MTT) (0.5 mg/mL) at 37 °C for 4 h. Then, 100 μL formazan dissolution was added to dissolve the formazan. Optical density (OD) values were measured at 570 nm using a microplate reader (Perkin-Elmer, Victor X4). The survival rate of the cells was calculated relative to the control cells. The half-maximal inhibitory concentration (IC_50_) was calculated by SPSS. All experiments were performed in triplicate.

### Cell apoptosis analysis

The number of apoptotic cells induced via CBD-Fab and NAT-Fab treatment was quantitated using an Annexin V-FITC/propidium iodide (PI) apoptosis assay kit (Cat. No 556547, BD Biosciences). A431 cells were incubated with 1.68 × 10^−4^ μM CBD-Fab and NAT-Fab for 48 h, and the cells were harvested and washed twice with cold PBS. The cells (1 × 10^5^ cells) were stained with 5 μL Annexin V-FITC and 5 μL PI for 30 min at room temperature in the dark. Finally, the cells were washed, resuspended in 400 μL of 1× binding buffer, and analyzed using flow cytometry. The apoptotic cells were expressed as a percentage of the total number of cells. All experiments were performed three times.

### EGF-R phosphorylation inhibition assay

A431 cells were seeded and plated onto 24-well plates, 10^5^ cells/well, in culture medium^39^. The cells starved overnight (culture medium containing 0.5% FBS) and incubated with 10^−1^ μM EGFR-specific mAbs (cetuximab, CBD-Fab, or NAT-Fab) for 30 min. Then, the cells were stimulated with EGF (20 ng/mL, Sigma, Shanghai, China) under serum starvation conditions for 15 min at 37 °C, 5% CO_2_. Cells were washed with ice-cold PBS and scraped immediately after adding 50 μL of radioimmunoprecipitation assay buffer (RIPA buffer) (Sigma, Shanghai, China). The protein extracts were separated by electrophoresis on 7.5% sodium dodecylsulfate-polyacrylamide gels (SDS-PAGE) and transferred to PVDF membranes by electroblotting. The membranes were processed for immunostaining with mouse monoclonal IgG1 anti-phospho-EGF-R Abs (Tyr1068, sc-377547, SantaCruz) and a mouse monoclonal IgG1 anti-EGF-R Abs (sc-377073, SantaCruz). The horseradish peroxidase-conjugated antibody (Sigma, Shanghai, China) was used as secondary antibody. At last, the bands were visualized with an electrochemiluminescence reagent (ECL, Beyotime, Haimen, China) and detected using a luminescent image analyzer LAS-4000 mini (Fujifilm, Tokyo, Japan).

### Mouse tumor xenograft models

Six- to eight-week-old female Nude BALB/c mice (nu/nu) (18 to 20 g) were purchased from Nanjing SIKERUI Biological Technology Co., Ltd. The mice were allowed to acclimatize to local conditions for 1 week prior to receiving injections of cancer cells. Mice were housed in air-filtered laminar flow cabinets and handled with aseptic procedures under a 12-hour light cycle, and food and water were provided ad libitum. The mice were checked at least twice per week for clinical signs of disease and discomfort. A431 cells (5 × 10^6^ cells) in the logarithmic growth phase were harvested and inoculated s.c. into the right flank of each mouse, as described in previous reports^40^,^41^. The mice were divided into the following dosing regimens: 1) On day 1 after tumor cells inoculation, the mice were randomly allocated to CBD-Fab, NAT-Fab, cetuximab, and Control groups (n = 5). Then, the mice were administered i.p. CBD-Fab, NAT-Fab, and cetuximab in a single treatment (15 μM in 450 μL PBS) on both day 1 and day 7; and 2) On day 10, when the tumors reached an average volume of 200 mm^3^, the animals were randomly divided into CBD-Fab, NAT-Fab, and cetuximab groups (n = 5), and the mice were injected i.p. with 15 μM CBD-Fab, NAT-Fab, and cetuximab in 450 μL PBS every two days for 4 weeks. The control mice were treated with the same volume of PBS. Tumor growth was assessed twice weekly via caliper measurement. Tumor volume (mm^3^) was calculated using the following formula: π/6 × larger diameter × (smaller diameter)^2^. The mouse experiments repeated twice. Also, the tumor tissues were cut up, washed with ice-cold PBS, and the total protein of tissues were extracted by adding 100 μL of RIPA buffer. The protein extracts were separated by electrophoresis on 7.5% SDS-PAGE and transferred to PVDF membranes by electroblotting. The membranes were processed for immunostaining with anti phospho-EGF-R Abs and a mouse monoclonal IgG_1_ anti-EGF-R Abs. The horseradish peroxidase-conjugated antibody was used as secondary antibody. At last, the bands were visualized with an ECL reagent and detected using a luminescent image analyzer LAS-4000 mini.

### The biodistribution of CBD-Fab and NAT-Fab *in vivo*

The *in vivo* targeting performance was assessed by quantitative biodistribution studies as described before (Zhang *et al.*) and the antibodies were labeled with Cy7^42^. Briefly, 20 mg of EDC(Sigma, Shanghai, China) was mixed with 100 mL of 50 mM phosphate buffer (pH 8.5) containing 2 mg of Cy7 (Carboxyl terminus, Fanbo Biochemicals Co. Ltd., Beijing) for 15 min of COOH activation, and then the antibody was added into the solution with a molar ratio at 20:1 (Cy7: antibody). The solution was further stirred at 4 °C for 4 h, before it was concentrated and rinsed with PBS through spin-dialysis (10 kDa, Millipore, Shanghai, China) to generate antibody- Cy7. Then, 15 μM antibody-Cy7 (CBD-Fab, cetuximab, or NAT-Fab) in 450 μL PBS was i.p. injected into the tumor-bearing mice. Mice were sacrificed at different time points after injection (2 h and 24 h). The animals were dissected directly after sacrifice, and the heart, liver, lungs, kidneys, spleen, and tumors were removed and spectrally imaged by the IVIS Lumina imaging system. The averaged fluorescence intensity of Cy7 of each imaged organ was calculated for a semi-quantitative biodistribution analysis.

### The sustained release of CBD-Fab *in vivo*

When the tumors reached an average volume of 200 mm^3^, the mice were treated with 15 μM CBD-Fab, NAT-Fab, or cetuximab in 450 μL PBS. Then, the mice were euthanized at different time points (2, 12, 24, 48, 72, and 96 h), and the tumors were harvested (n = 5). The tumor cells were detached using enzyme-free cell dissociation buffer (Life Technologies, Shanghai, China) with 0.03% collagenase (Life Technologies, Shanghai, China) at 37 °C for 15 min. After washing five times with PBS containing 5% FBS, the tumor cells (1 × 10^6^ cells) were incubated with anti-His antibody at 4 °C for 2 h, and anti-mouse IgG_1_-FITC was added as a secondary antibody at 37 °C for 30 min. Finally, the cells were analyzed using flow cytometry^10^. All experiment was performed three times. Cryosections (7-μm-thick) from the same tumor xenografts were fixed in acetone for 15 min. The sections were stained to detect the retention time of CBD-Fab, NAT-Fab, and cetuximab in tumors. For immunostaining, the anti-His antibody and anti-type I collagen antibody were used as a primary antibody, anti-mouse FITC-IgG_1_ and anti-rabbit IgG Fc (DyLight® 594) were used as the secondary antibody, and the nucleus was labeled with DAPI. The sections were observed under an Olympus BX-51 light microscope (Olympus, Tokyo, Japan) and analyzed with Image-Pro Plus analysis software (Media Cybernetics, Inc., Silver Spring, MD, USA).

### Immunohistochemistry

Adjacent sections were stained with Masson’s trichrome staining to visualize collagen in tumors. On day 30, the mice were immediately euthanized and their tumors were excised. Tumor tissues were fixed in 4% paraformaldehyde and embedded in paraffin blocks. The sections (4-μm-thick) were cut and stained with H&E and Masson’s trichrome. Then, the sections were observed under an Olympus BX-51 light microscope.

For immunohistochemical analysis, the tumor tissues were cut into 7-μm-thick sections and fixed in acetone for 15 min. The sections were incubated using the following primary antibodies: CD31 (1:200 dilution, sc-53411, Santa Cruz Biotechnology, Inc.) and Ki67 (1:200 dilution, sc-101861, Santa Cruz Biotechnology, Inc.). Anti-mouse FITC-IgG_1_ and Alexa Fluor 594 donkey anti-mouse IgG (H + L) were used as secondary antibodies, respectively. TdT-UTP transferase nick-end labeling (TUNEL) assays were performed using the one step TUNEL kit (Beyotime Institute of Biotechnology, Haimen, China) following the manufacturer’s instructions. The nuclei were labeled with DAPI. All sections were observed under an Olympus BX-51 light microscope and analyzed with Image-Pro Plus analysis software.

### Statistical analysis

The data analysis was performed using GraphPad Prism 5.0 and Statistical Package for the Social Sciences (SPSS) statistical software (SPSS Inc., Chicago, IL, USA). All data are presented as the mean ± SD. Differences between groups were analyzed using one-way ANOVA, and *p* < 0.05 was considered statistically significant.

### Study approval

All animal studies were permitted by the Institutional Animal Care and Use Committee of the Suzhou Institute of Nano-Tech and Nano-Bionics (SINANO), Chinese Academy of Sciences and were conducted in compliance with its recommendations.

## Additional Information

**How to cite this article**: Liang, H. *et al.* A collagen-binding EGFR antibody fragment targeting tumors with a collagen-rich extracellular matrix. *Sci. Rep.* **5**, 18205; doi: 10.1038/srep18205 (2016).
